# Flea beetles of the West Indies: the genus *Hemilactica* Blake, 1937 (Coleoptera, Chrysomelidae, Galerucinae, Alticini)

**DOI:** 10.3897/zookeys.1044.62632

**Published:** 2021-06-16

**Authors:** Alexander S. Konstantinov

**Affiliations:** 1 Systematic Entomology Laboratory, USDA, c/o Smithsonian Institution, National Museum of Natural History, P. O. Box 37012, Washington, DC 20013-7012, USA Smithsonian Institution Washington United States of America

**Keywords:** Beetle diversity, Dominican Republic, lectotype designation, Neotropical Region, new species

## Abstract

The West Indian flea beetle genus *Hemilactica* Blake, 1937 is reviewed. Two new species, both from the Dominican Republic are described and illustrated: *H.erwini***sp. nov.** and *H.sierramatringarcia***sp. nov.** In addition, images of the holotypes of *H.portoricensis* Blake, *H.pulchella* Blake, and *H.rugosa* Blake are provided. *Lacticamegaspila* (Blake) is transferred to *Hemilactica*. A lectotype of *H.quatuordecimpunctata* (Suffrian, 1868) is designated and illustrated, and a key to the *Hemilactica* species and a key for identification of *Hemilactica* and related genera occurring in the Western Hemisphere are provided.

## Introduction

As of the most recent account, there are approximately 10,000 valid flea beetle species (Coleoptera: Chrysomelidae: Galerucinae: Alticini) assigned to 599 valid genera in the World. These constitute the most species-rich family level taxon in leaf beetle family and of these, 59 valid genera and 384 valid species are known to occur in the West Indies. Seventeen genera are West Indian endemics, including *Hemilactica* Blake, 1937. The genus contains nine species, with seven species known from Cuba, one from Puerto Rico, and one from the Dominican Republic. Two new species have been discovered in the Dominican Republic. They are described below.

## Materials and methods

Dissecting techniques and morphological terminology follow Konstantinov (1998). Specimen observations were made with a Zeiss Stemi SV11 Apo microscope. Digital photographs of morphological structures were taken with Axio Zoom V16 microscope and AxioCam HRC digital camera attached to it and with AxioCam HRC Zeiss attached to Leitz Diaplan compound microscope. Additional images were taken with Macropod Pro photomacrography system (Macroscopic Solutions, LLC, Tolland, CT, USA). Specimen labels are cited verbatim, according to the format justified previously (Konstantinov 1998; Konstantinov and Lingafelter 2002; Konstantinov et al. 2011).

The specimens are deposited in collections of the National Museum of Natural History, Smithsonian Institution, Washington DC, USA (**USNM**); Florida State Collection of Arthropods, Tallahassee, FL, USA (**FSCA**); and Museo Nacional de Historia Natural, Santo Domingo, Dominican Republic (**MHND**); Museum of Comparative Zoology, Harvard University, Cambridge, MA USA (**MCZC**), and Martin-Luther-Universität, Zentralmagazin Naturwissenschaftlicher Sammlungen, Zoologische Sammlung, Germany (**MLUH**).

## Taxonomy

### Hemilactica

Taxon classificationAnimaliaColeopteraChrysomelidae

Blake, 1937

BD90C768-1C94-5182-BC8D-A39AE186B399

Hemilactica Blake, 1937: 37. Type species: Hemilacticapulchella Blake, 1937: 37, by original designation.

#### Distribution.

Cuba, Hispaniola, Puerto Rico.

#### Host plants.

*Micropholisguyanensis* (A. DC.) Pierre (Sapotaceae), wild balata (Blake 1964 for *Hemilacticaportoricensis* Blake).

#### Remarks.

While describing the genus, Blake compared it with *Lactica* Erichson and *Diphaulaca* Chevrolat. It is indeed similar externally to both. However, *Hemilactica* specimens are missing sclerotized vaginal palpi as *Lactica* and related genera (Viswajyothi and Konstantinov 2020). The type species of *Diphaulaca* [*D.aulica* (Olivier)] has vaginal palpi well sclerotized and fully visible. Because of the structure of on the beetle’s head sulci and ridges, the grooves on the pronotum and general body shape, *Hemilactica* generally fits into the *Monomacra* group of genera as roughly defined by Bechyne and Springlova de Bechyne (1975) and described in more details in Viswajyothi and Konstantinov (2020). In order to facilitate identification of *Hemilactica*, a key to it and related genera previously published (Viswajyothi and Konstantinov 2020) is provided at the end of the paper.

Currently known *Hemilactica* species exhibit some noticeable differences in “genus” level characters as they are currently understood for the purpose of revising flea beetle genera of the West Indies. The type species, *H.pulchella* and *H.rugosa* Blake are quite similar in having strongly punctate dorsum and relatively long and narrow frontal ridge, while species that Blake (1964) described later (e.g., *H.portoricensis*) have a much shorter and wider frontal ridge and smooth elytra with much smaller elytral and pronotal punctations. However, the other substantial features of these beetles look similar. Therefore, they are all retained under *Hemilactica* until more evidence comes to light. *Lacticamegaspila* Blake (Fig. 26) is clearly congeneric with *Hemilacticaportoricensis* and therefore is transferred here to *Hemilactica*.

### Hemilactica erwini sp. nov.

Taxon classificationAnimaliaColeopteraChrysomelidae

8A900F0A-1804-5782-9382-E0A1BE33C37B

http://zoobank.org/2C19CB9E-F020-4011-9D4B-6B83A4FEB2CD

Figures 1 , 2–5 , 6–11

#### Material examined.

***Holotype***, male. Labels: 1) Dominican Rep.: Prov. Barahona, nr. Filipinas, Larimar Mine: 20–26.VI.1992; R. E. Woodruff & P. E. Skelley, at night; 2) ***Holotype****Hemilacticaerwini* des. A. Konstantinov 2020 (FSCA). ***Paratypes*** with the same labels as holotype (1 FSCA, 2 USNM). Paratype with the same labels as holotype except 26.VI. (FSCA). ***Paratype*** female. Labels: 1) Dominican Republic: Independencia Prov., PN Sierra de Baoruco, (S of Puerto Escondido), 15.VII.04, 1215–400 m, 18°16.035'N, 71°32.684'W, leg. A. Konstantinov; 2) ***Paratype****Hemilacticaerwini* des. A. Konstantinov 2020 (USNM).

#### Diagnosis.

Pronotum with two longitudinal dark spots on both sides of middle. Elytron with following dark spots: one on humeral callus, one medially to it, on basal callus, one below basal callus towards middle of elytron, one laterally towards side of elytron. Spots vary in size and color, some barely visible. Supracallinal sulcus poorly developed, straight, or convex, perpendicular to midline. Frontal ridge relatively long, dorsally wider than ventrally. Receptacle of spermatheca with inner side straight, outer side convex. Median lobe of aedeagus in lateral view bends abruptly about middle, with tip curving dorsally. Median lobe in ventral view more or less parallel sided basally, narrowing gradually towards narrow apex, lacking denticle.

**Figure 1.**
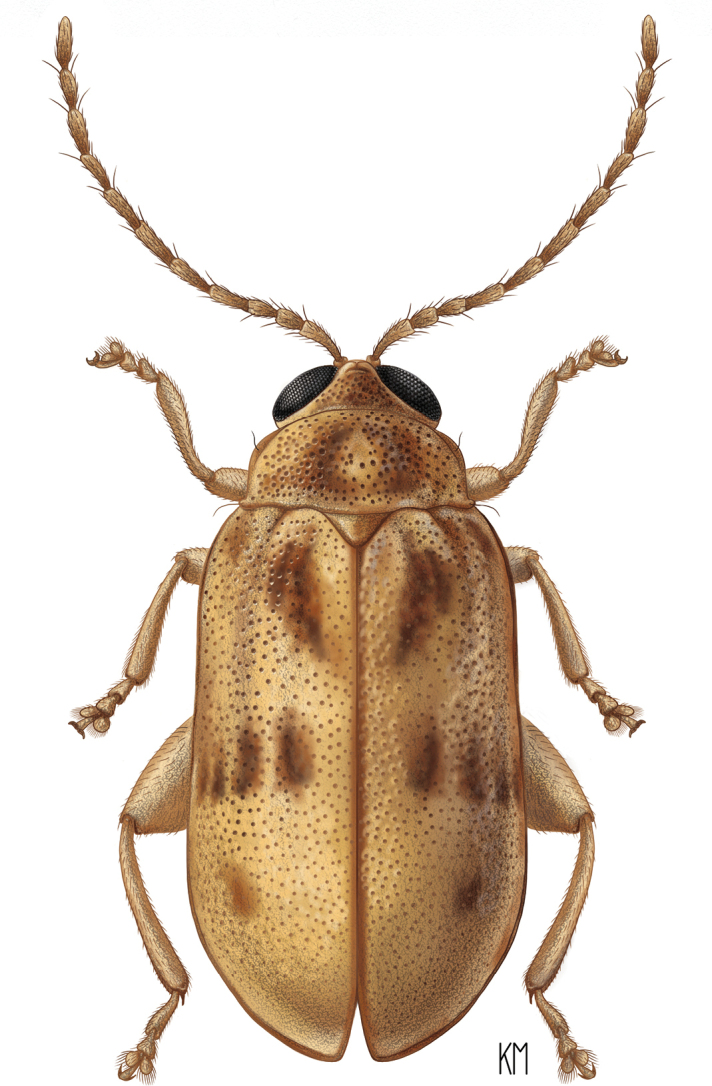
Adult *Hemilacticaerwini* sp. nov., illustration by Katy Marchese (Systematic Entomology Laboratory internship program 2017).

#### Description.

**Figures 2–5.**
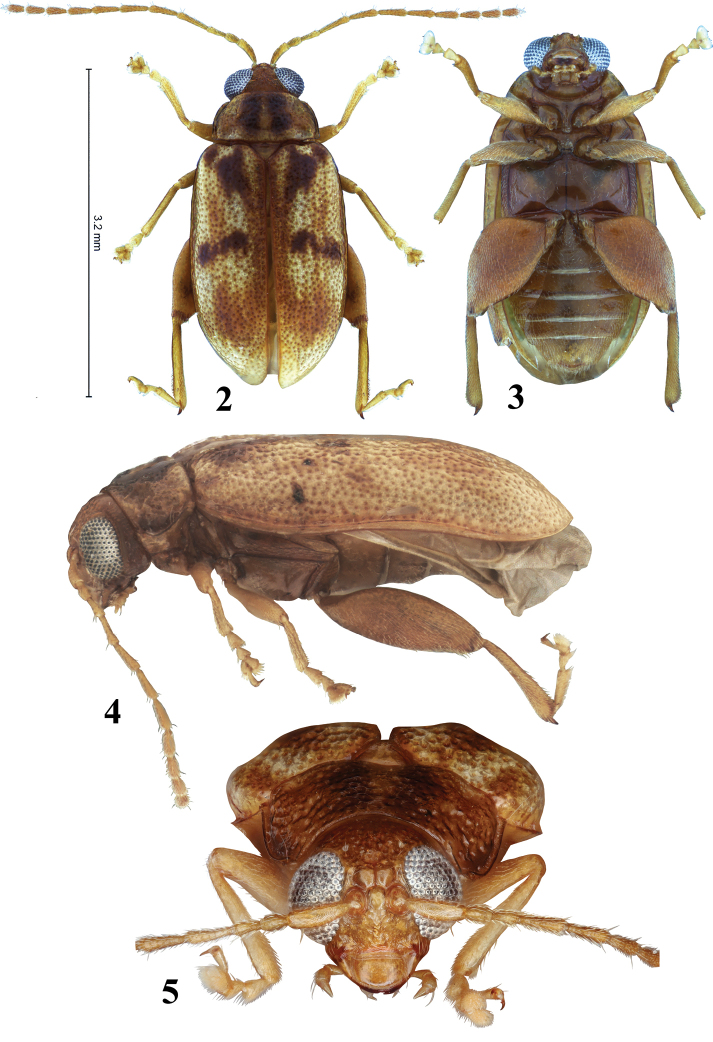
*Hemilacticaerwini* sp. nov. **2** dorsal habitus **3** ventral habitus **4** lateral habitus **5** frontal habitus.

***Body*** length 3.02–3.29 mm. Body width (widest point of elytra) 1.56–1.62 mm. Body height 1.08–1.13 mm. Pronotum and elytron yellowish, straw color with poorly defined, brownish spots. Pronotum with two longitudinal spots on both sides of middle. Elytron with following spots: one on humeral callus, one medially to it, on basal callus, one below basal callus towards middle of elytron, one laterally towards side of elytron. Spots vary in size and color, some barely visible (Fig. 2).

***Head*.** Surface of vertex densely and evenly covered with large punctures (Fig. 5). Orbit reduced to a narrow grove between eye and antennal callus. Supraorbital pore well developed, noticeable among other punctations. Inner margins of eyes slightly concave to straight, diverging towards mouth parts. Distance between eyes above antennal sockets in frontal view slightly greater than transverse diameter of eye. Sides of head below eyes converging ventrally. Anterior margin of labrum entire. Labrum with two pairs of setae placed symmetrically on sides. Midcranial suture absent. Supraorbital sulcus represented by fold between antennal callus and orbit. Orbital sulcus well developed. Supracallinal sulcus poorly developed, straight, or convex, perpendicular to midline. Supracallinal and supraorbital sulci form wide angle. Midfrontal sulcus well developed, long. Suprafrontal sulcus well developed, antennal calli and top of frontal ridge meet, separated by groove. Antennal calli nearly trapezoidal or nearly quadrate, directed longitudinally, not entering interantennal space. Frontal ridge relatively long, dorsally wider than ventrally. Its sides between antennal sockets slightly concave. Dorsal side of frontal ridge acute. Frontal ridge extends slightly between antennal calli. Anterofrontal ridge very low, merges with clypeus.

***Antenna*** filiform, reaching beyond half elytron (Fig. 2). Antennomere 1 shorter than next two antennomeres combined. Antennomere 2 elongate, shorter than 3, longer than half of it, narrower than antennomere 1, wider than antennomere 3. Antennomere 3 shorter than 4. Antennomere 5 shorter than 4 and as long as 6. Antennomeres 6 and 7 nearly as wide as antennomeres 4 and 5 separately. Antennomere 7 slightly narrower than 8.

**Figures 6–11.**
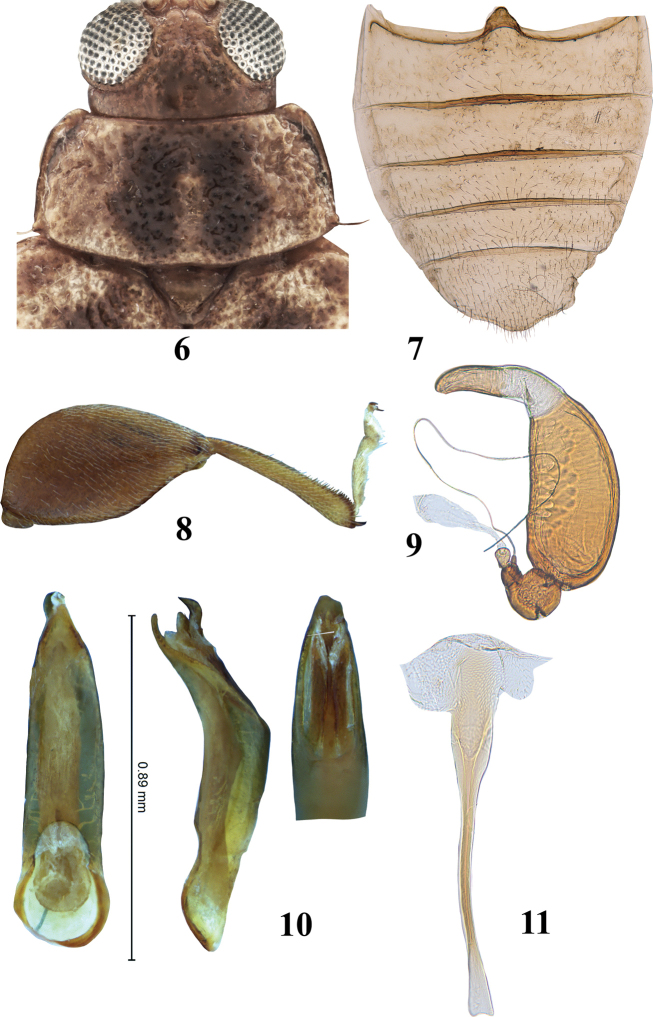
*Hemilacticaerwini* sp. nov. **6** pronotum **7** abdominal ventrites **8** hind leg **9** spermatheca **10** median lobe of aedeagus (ventral, lateral, and dorsal views) **11** tignum.

***Prothorax*** surface glabrous, deeply and coarsely punctate (Fig. 6). Anterolateral callosity elongate, not expanded beyond lateral margin, facing anterolaterally. Anterior setiferous pore along anterolateral callosity situated close to posterior end. Anterolateral corners of pronotum projected slightly forward. Sides of pronotum slightly and evenly convex more so anteriorly. Base of pronotum with two short impressions visible only near basal margin. Pronotal base evenly convex. Antebasal transverse impression on pronotum shallow and poorly defined, better visible near longitudinal impressions, limited by them. Posterolateral callosity situated on corner of posterior and lateral margins. Procoxal cavities open. Intercoxal prosternal process convex at apex, extends beyond procoxae.

***Elytra*** at base wider than base of pronotum, with convex sides. Humeral and basal calli present. Elytral punctation deep, coarse, and confused. Ridges on elytra absent (Fig. 1).

***Legs*.** Pro- and mesotibiae without apical spur and with longitudinal ridge. Protarsomere 1 in males wider and longer than in females. Metatibia (Fig. 8) straight in dorsal view, slightly curved in lateral view, more or less cylindrical around middle. Metatibia on lateral side without small denticles. Metatibial apex flattened dorsally before tarsal insertion. Metatibial spur simple, narrow, ending in one tooth, situated laterally, nearly as long as greatest width of metatibial apex. Incision of metatarsomere 3 present. Claw appendiculate with a short lobe.

***Genitalia*.** Spermatheca (Fig. 9) with receptacle and pump with distinct border in between. Receptacle longer than wide, in a single plane, inner side straight, outer side convex, longer and wider than pump. Pump more or less straight. Duct of spermatheca without coils, roundish, narrowing abruptly towards gland. Vaginal palpi absent. Tignum narrow anteriorly into a narrow lobe (Fig. 11). Median lobe of aedeagus (Fig. 10) bends abruptly about middle, with tip curving dorsally in lateral view. In ventral view more or less parallel-sided basally, narrowing gradually towards narrow apex, lacking denticle.

#### Habitat.

Seasonally dry tropical forest.

#### Etymology.

This species is named after Terry L. Erwin, USNMColeoptera curator, prolific ground beetle systematist, and pioneering scholar of tropical biodiversity.

#### Comments.

*Hemilacticaerwini* is similar to the type species of the genus, *H.pulchella* Blake and *H.rugosa* Blake in having relatively narrow frontal ridge and deeply and coarsely punctate elytra with brownish, poorly defined spots and lacking ridges. It may be separated from them by the smaller, less differentiated and paler spots on pronotum and wider tip of the median lobe of the aedeagus. *Hemilacticaerwini* is easily distinguished from the rest *Hemilactica* species as they have relatively small elytral and pronotal punctations, elytral surface shiny with bright blue or black spots and longitudinal ridges. In addition, *H.erwini* may be identified with the help of the key below.

### Hemilactica sierramartingarcia sp. nov.

Taxon classificationAnimaliaColeopteraChrysomelidae

B110B55A-59F3-5230-B481-9EF944D69B09

http://zoobank.org/4122BF64-1C7D-430D-8568-7386E2D9E59F

Figures 12 , 13–16 , 17–24

#### Type material examined.

***Holotype***, male. Labels: 1) Dominican Republic, Barahona Pr., Sierra Martin Garcia 9.XII.2014, 925 m, WP-511, 18°21.224'N, 71°00.870'W Leg. A. S. Konstantinov; 2) ***Holotype****Hemilacticasierramartingarcia* des. A. Konstantinov 2020 (USNM). ***Paratypes*** with the same labels as holotype (5 USNM, 2 MHND).

#### Diagnosis.

Pronotum, thorax, antennae, and legs uniformly orange, with tips of legs a bit darker. Elytra uniformly blue. Supracallinal sulcus poorly developed, straight, or convex, perpendicular to midline. Midfrontal sulcus visible, long, but weakly impressed. Frontal ridge relatively long, dorsally wider than ventrally. Median lobe of aedeagus bends gradually about middle, with tip curving dorsally in lateral view. Spermathecal pump more or less straight, wider than receptacle, with small round structure at the tip.

**Figure 12.**
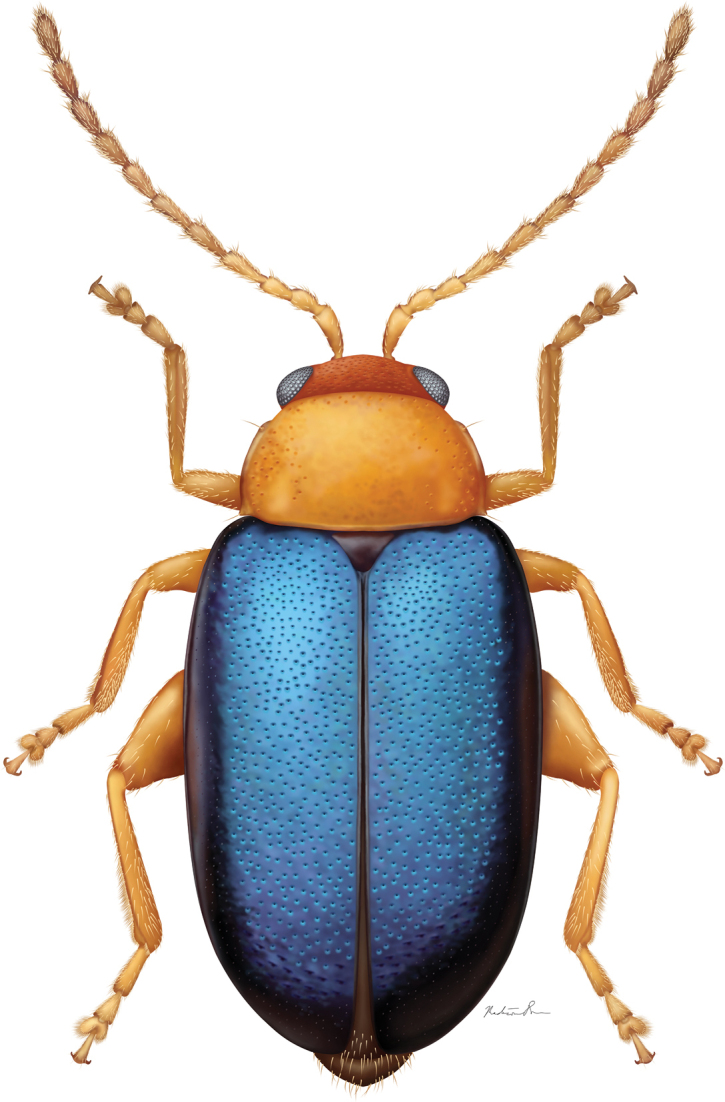
*Hemilacticasierramartingarcia* sp. nov., illustration by Madison Dorr (Systematic Entomology Laboratory internship program 2020).

#### Description.

***Body*** length 2.16–2.70 mm. Body width (widest point of elytra) 1.13–1.51 mm. Body height 0.81–0.86 mm. Pronotum, thorax, antennae, and legs uniformly orange, with tips of legs a bit darker. Elytra uniformly blue. Abdomen dark brown with tip a bit lighter.

***Head*.** Surface of vertex densely and evenly covered with large punctations (Fig. 15). Orbit narrow. Supraorbital pore well developed, noticeable among other punctations. Inner margins of eyes slightly concave to straight, diverging towards mouth parts. Distance between eyes above antennal sockets in frontal view three times greater than transverse diameter of eye. Sides of head below eyes converging ventrally. Anterior margin of labrum entire. Labrum with two pairs of setae placed symmetrically on sides of labrum. Midcranial suture absent. Supraorbital sulcus represented by fold between antennal callus and orbit. Orbital sulcus poorly developed. Supracallinal sulcus poorly developed, straight, or convex, perpendicular to midline. Supracallinal and supraorbital sulci form wide angle. Midfrontal sulcus visible, long, but weakly impressed. Suprafrontal sulcus long, antennal calli and top of frontal ridge meet, separated by groove. Antennal calli nearly trapezoidal or nearly quadrate, directed longitudinally, not entering interantennal space. Frontal ridge relatively long, dorsally wider than ventrally. Its sides between antennal sockets slightly convex. Dorsal side of frontal ridge acute. Frontal ridge extends slightly between antennal calli. Anterofrontal ridge very low, merges with clypeus.

**Figures 13–16.**
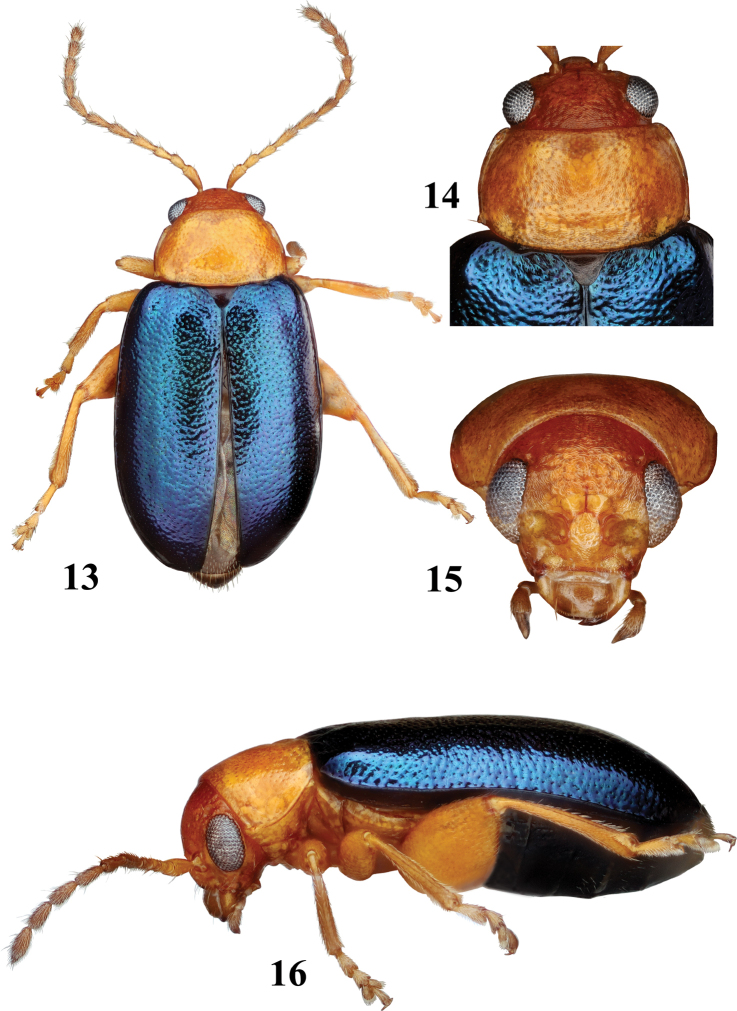
*Hemilacticasierramartingarcia* sp. nov. **13** dorsal habitus **14** pronotum **15** head, frontal view **16** lateral habitus.

**Figures 17–24.**
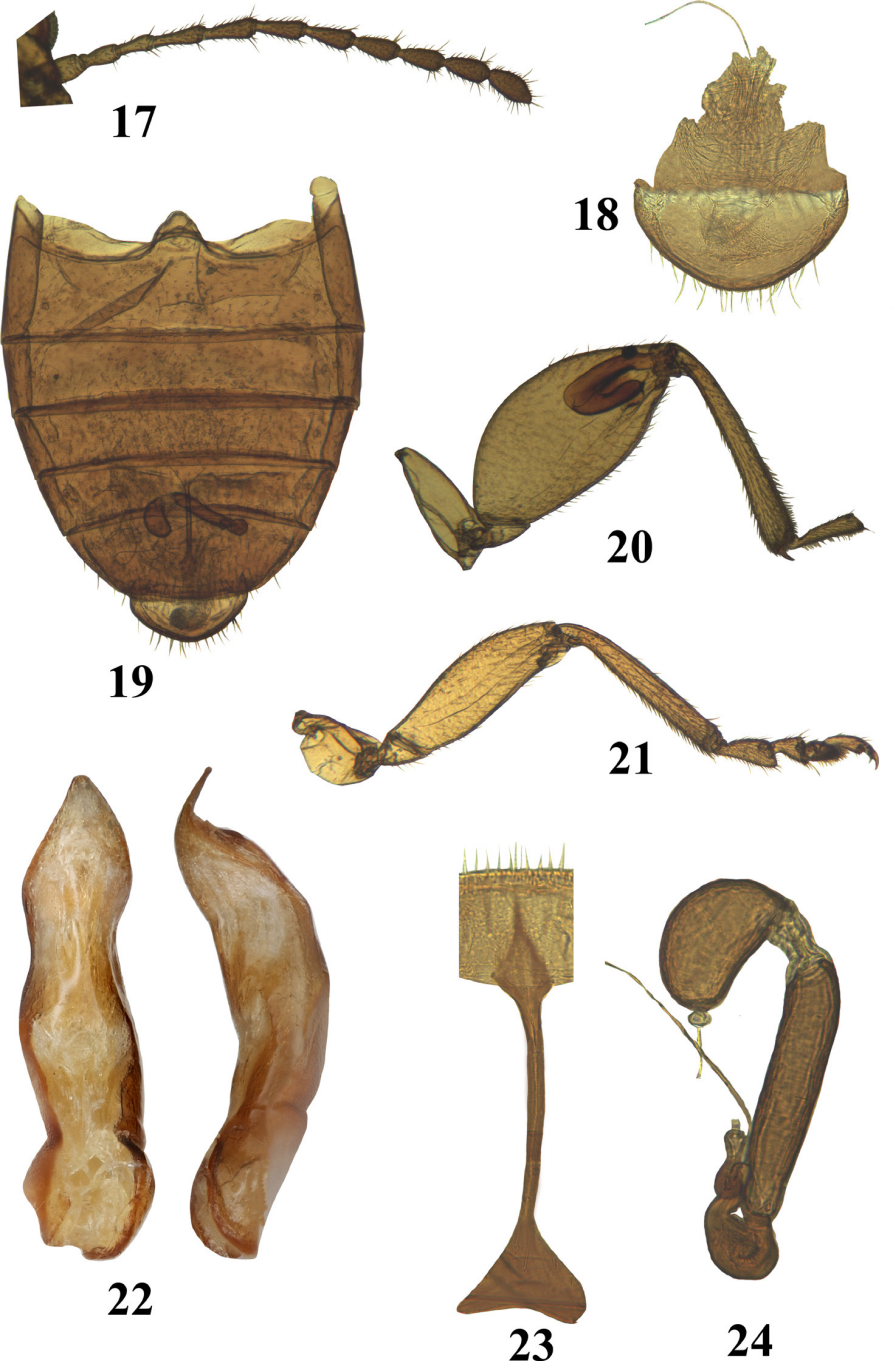
*Hemilacticasierramartingarcia* sp. nov. **17** antenna **18** last abdominal tergite and vagina **19** abdominal ventrites **20** hind leg **21** front leg **22** median lobe of aedeagus (ventral, lateral, and dorsal views) **23** tignum **24** spermatheca.

***Antenna*** filiform (Fig. 17), reaching beyond half elytron. Antennomere 1 shorter next two antennomeres combined. Antennomere 2 elongate, as long as 3, narrower than antennomere 1, wider than antennomere 3. Antennomere 3 shorter than 4. Antennomere 5 as long as 4 and longer than 6. Antennomeres 6 and 7 nearly as wide as antennomeres 4 and 5 separately. Antennomere 7 approximately as narrow as 8.

***Prothorax*** surface glabrous (Fig. 14), covered with relatively shallow, sparsely placed punctations. Anterolateral callosity elongate, not expanded beyond lateral margin, facing anterolaterally. Anterior setiferous pore along anterolateral callosity situated close to posterior end. Anterolateral corners of pronotum projected slightly forward. Sides of pronotum slightly and evenly convex. Base of pronotum with two short impressions visible only near basal margin. Pronotal base evenly convex. Antebasal transverse impression on pronotum shallow and poorly defined, better visible near longitudinal impressions, limited by them. Posterolateral callosity situated on corner of posterior and lateral margins. Procoxal cavities open. Intercoxal prosternal process convex at apex, extends beyond procoxae.

***Elytra*** at base wider than base of pronotum, with convex sides. Humeral and basal calli present. Elytral punctation confused. Punctations deeper and slightly larger than those of pronotum. Ridges on elytra absent (Fig. 13).

***Legs*.** Pro- and mesotibiae without apical spur and with longitudinal ridge (Fig. 21). Protarsomere 1 in males wider and longer than in females. Metatibia straight in dorsal view, slightly curved in lateral view, more or less cylindrical around middle. Metatibia on lateral side without small denticles (Fig. 20). Metatibial apex flattened dorsally before tarsal insertion. Metatibial spur simple, narrow, ending in one tooth, situated laterally, nearly as long as greatest width of metatibial apex. Incision of metatarsomere 3 present. Claw appendiculate with a short lobe.

***Genitalia***: Spermatheca with receptacle and pump with distinct border in between (Fig. 24). Receptacle longer than wide, in a single plane, inner side straight, outer side convex, longer and narrower than pump. Pump more or less straight, wider than receptacle, with small oval structure at the tip. Duct of spermatheca without coils, roundish, narrowing abruptly towards gland. Vaginal palpi absent. Tignum widens anteriorly into a wide lobe (Fig. 23). Median lobe of aedeagus in lateral view bends gradually at about middle, with tip curving dorsally. In ventral view slightly constricted basally and above middle basally, lacking denticle (Fig. 22).

#### Habitat.

Seasonally dry tropical forest (Franklin et al. 2019).

#### Etymology.

Specific epithet is a noun in apposition based on the type locality, Sierra Martin Garcia.

#### Comments.

*Hemilacticasierramartingarcia* is quite unusual among *Hemilactica* species because its uniformly blue elytra. Among them it is similar to *H.stomachosa* (Suffrian), which elytra are also bluish. The concept of *H.stomachosa* is based on the specimen from Cuba that Blake identified as such with “?”. Both species may be separated by weakly developed pronotal grooves in *H.sierramartingarcia*. Pronotal grooves are well developed in *H.stomachosa*. *Hemilacticasierramartingarcia* is similar to the type species of the genus, *H.pulchella* Blake, *H.erwini*, and *H.rugosa* Blake in having deeply and coarsely punctate and lacking elytral ridges. Interestingly, median lobe of aedeagus in *H.sierramartingarcia* and in *H.erwini* have an elongate apex strongly bent dorsally (Figs 10, 22), which may be a character that identifies the genus. *Hemilacticasierramartingarcia* may be separated from them by the smaller size and the body color. In addition, *H.sierramartingarcia* may be identified with the help of the key below.

### Key for the identification of *Hemilactica* species

**Table:** 

1	Elytra uniformly blue	**2**
–	Elytra yellow with brown, blue, and blackish spots	**3**
2	Pronotum with barely visible longitudinal and transverse impressions. Elytron even (Fig. 13)	***Hemilacticasierramartingarcia* sp. nov.**
–	Pronotum with well developed, well visible longitudinal and transverse impressions. Elytron with poorly developed longitudinal ridges	***Hemilacticastomachosa* (Suffrian)**
3	Elytron yellowish, dull, with large and deep punctations and brown spots, mostly lacking ridges (Fig. 2)	**4**
–	Elytron yellowish, shiny, with small and shallow punctations and often with blue or blackish spots, with ridges (Figs 25, 26)	**7**
4	Elytron with two brown transverse zigzagged lines and lighter color in between. Elytron apex lighter in color than elytral disc	***Hemilacticagraphica* Blake**
–	Elytron with different pattern. Elytral apex as light in color as elytral disc	**5**
5	Elytron with eight dark brown spots: one on basal callus, one on humeral callus, three spots across elytron near middle, and three above elytral apex (Fig. 29)	***Hemilacticaquatuordecimpunctata* (Suffrian)**
–	Elytron with different pattern	**6**
6	Most of pronotum dark brown. Apex of median lobe of aedeagus produced into a long, thin projection slightly widening at the apex (Fig. 30)	***Hemilacticarugosa* Blake**
–	Most of pronotum yellowish. Apex of median lobe of aedeagus produced into a relatively short projection not widening at the apex (Fig. 2)	***Hemilacticaerwini* sp. nov.**
7	Elytron dark yellow to orange with wide, bright greenish blue bands basally and apically (Fig. 25)	***Hemilacticaclara* Blake**
–	Elytron with different pattern	**8**
8	Elytron dark bluish with pale margin and pale band across middle	***Hemilacticacrucifera* Blake**
–	Elytron yellowish with or without various dark spots	**9**
9	Pronotum and elytra entirely yellowish to orange, lacking markings, except bases of elytra slightly darker (Fig. 26)	***Hemilacticamegaspila* (Blake)**
–	Elytron with various dark spots	**10**
10	Elytron with multiple longitudinal ridges and merging dark spots at base in middle and apex (Fig. 28)	***Hemilacticapulchella* Blake**
–	Elytron with large ridge and two, bright purple large spots, basally and apically	**11**
11	Front and mid tibiae yellow. Basal and apical elytral spots more or less roundish (Fig. 27)	***Hemilacticaportoricensis* Blake**
–	Front and mid tibiae dark. Basal and apical elytral spots with more or less straight margin, apical spot in particular	***Hemilacticafasciata* Blake**

**Figures 25–28.**
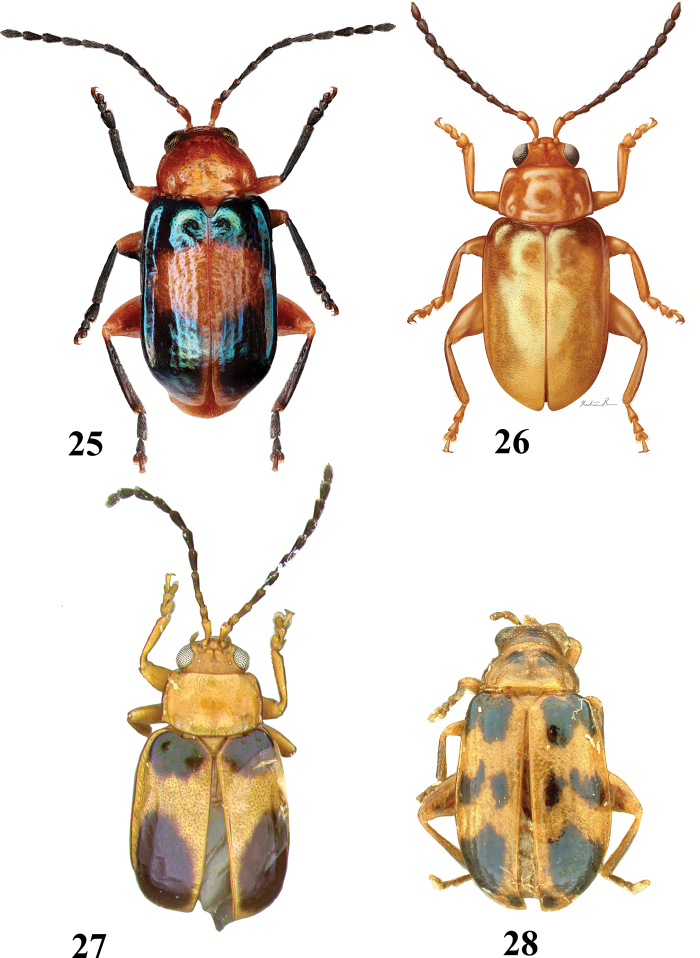
Adult *Hemilactica* species dorsal habitus **25***H.clara***26***H.megaspila*, illustration by Madison Dorr (Systematic Entomology Laboratory internship program 2020) **27***H.portoricensis*, holotype **28***H.pulchella*, holotype.

### Species list

**1. *Hemilacticaclara*Blake 1959: 244 (type locality: La Brena, Moa, Oriente Province, Cuba, holotype; male, type depository: USNM).**

**Distribution.** Cuba.

**Material examined.** Pilote, Moa.-Ote. Junio 1954. Zayas-Alayo coll.; ***Paratype*** No. 64663, USNM; *Hemilacticaclara* Blake (7 exx, USNM).

**2. *Hemilacticacrucifera*Blake 1959: 245 (type locality: Sierra del Cristol, Oriente Province, Cuba; holotype; male; type depository: Zayas Collection, Cuba).**

**Distribution.** Cuba.

**3. *Hemilacticaerwini* sp. nov. (type locality: Prov. Barahona, nr. Filipinas, Larimar Mine; holotype, male; type depository: FSCA).**

**Distribution.** Hispaniola: Dominican Republic.

**Material examined. *Holotype***, male. Dominican Rep.: Prov. Barahona, nr. Filipinas, Larimar Mine: 20–26.VI.1992; R. E. Woodruff & P. E. Skelley, at night (FSCA). Paratypes with the same labels as holotype (1 FSCA, 2 USNM). ***Paratype*** with the same labels as holotype except 26.VI.1992 (FSCA). Paratype female. Dominican Republic 15.VII.04 Independencia Prov., PN Sierra de Baoruco, (S of Puerto Escondido), 1215–400 m, 18°16.035'N, 71°32.684'W, leg. A. Konstantinov (USNM).

**4. *Hemilacticafasciata*Blake 1938: 50 (type locality: Upper Ovando R, eastern Oriente Province, Cuba; holotype; male; type depository: MCZC).**

**Distribution.** Cuba.

**5. *Hemilacticagraphica*Blake 1939: 233 (type locality: Dominican Republic: Mt. Diego de Ocampo; holotype; female; type depository: MCZC).**

**Distribution.** Hispaniola: Dominican Republic.

**6. *Hemilacticamegaspila* (Blake) 1948: 144 (original genus: Lactica; type locality: Villalba, Puerto Rico; holotype; male; type depository: MCZC). New Combination.**

**Distribution.** Puerto Rico.

**Material examined.** Puerto Rico: ElYunque, El, Toro trail WP-230 N18.16.332 W65.49.753, h = 1066 m, 16.VI.2008, leg. A. Konstantinov (5 exx USNM).

**7. *Hemilacticaportoricensis*Blake 1964: 20 (type locality: Mutrullas, Puerto Rico; holotype; male; type depository: USNM).**

**Distribution.** Puerto Rico.

**Host plant.***Micropholisguyanensis* (A. DC.) Pierre (Sapotaceae), wild balata (Blake 1964).

**Material examined.** On tree at Villalba P.R., Jun. 18.1934, ss# 5656, RG Oakley; unknown tree, Ins. Gov. Finca Villalba P. R. Coll. 18 June 34, R. G. Oakley; USNM, ***Paratype***, 66190; *Hemilacticaportoricensis* Blake (4 USNM). on *Micropholiscurvata*, Matrullas P.R. Oct. 15. 34, SJ # 5841, RG Oakley; USNM, Paratype, 66190 (USNM).

**8. *Hemilacticapulchella*Blake 1937: 73 (type locality: Jarahueca, Oriente Province, Cuba; holotype; male; type depository: USNM).**

**Distribution.** Cuba.

**Material examined.** Jarahueca, Ote., Cuba, Jul. 14–18/27; S. C. Bruner; U.S.N.M, Paratype No. 51835; *Hemilacticapulchella* Blake m 41 (USNM).

**9. *Hemilacticaquatuordecimpunctata* (Suffrian) 1868: 206 (original genus: *Haltica* ; type locality: Cuba; lectotype, designated here; female; type depository: MLUH).**

### Distribution.

Cuba.

**Material examined. *Lectotype***, female. 31596; MLU Halle, WB Zoologie S.-Nr.7/1/10, T.-Nr. Haltica quatuordecimpunct.; ***Lectotype****Hemilacticaquatuordecimpunctata* (Suffrian) 1868 des. A.Konstantinov 2018 (1 MLUH).

**10. *Hemilacticarugosa*Blake 1937: 74 (type locality: Palma Mocha, Sierra Maestra, Oriente Provine, Cuba; holotype; male; type depository: USNM).**

**Distribution.** Cuba.

**Material examined.** Palma Mocha Mt.., S. Maestra, Cuba, May 16/48. J. Acuna, 3900–4500 ft.; *Hemilacticarugosa* (USNM). Sierra Maestra, Cuba. Julio 10–12 de 1922. Col. C. H. Ballou y S.C. Bruner 900–1000M; Hemilacticarugosa Blake; Type No 51836, U.S.N.M. (USNM)

**11. *Hemilacticasierramartingarcia* sp. nov. (type locality: Dominican Republic, Barahona Pr., Sierra Martin Garcia; holotype; male; type depository: USNM).**

**Distribution.** Hispaniola: Dominican Republic.

**Material examined. *Holotype*** male. Dominican Republic, Barahona Pr., Sierra Martin Garcia, 9.XII.2014, 925 m, WP-511, 18°21.224'N, 71°00.870'W Leg. A. S. Konstantinov; ***Holotype****Hemilacticasierramartingarcia* des. A. Konstantinov 2020 (USNM). Paratypes with the same labels as holotype (5 USNM, 2 MHND).

**12. *Hemilacticastomachosa* (Suffrian) 1868: 204 (original genus: *Haltica* ; type locality: Cuba; syntype; unknown; type depository: unknown).**

**Distribution.** Cuba, Pinar del Rio Province.

**Material examined.** Pan de Guajaibon, Prov. P. d. 1210 May 53 Zayas; Hemilactica?stomachosa Suffr (USNM).

### Key for identification of *Hemilactica* and related genera occurring in the Western Hemisphere

**Table:** 

1	Pronotum with a complex sculpture consisting of two longitudinal and two transverse ridges that connect to each other. Elytron with more than one longitudinal ridge. Dorsal surface covered with waxy substance	***Myrmeconycha* Konstantinov & Tishechkin**
–	Pronotum without two longitudinal and two transverse ridges that connect to each other. Elytron without longitudinal ridges, or with only one ridge. Dorsal surface not covered with waxy substance	**2**
2	Base of pronotum without transverse or longitudinal impressions	**3**
–	Base of pronotum with transverse or longitudinal impressions or both	**4**
3	Antennomeres beyond second cylindrical, antennomeres 4 and 5 not wider than apical antennomeres	***Disonycha* Chevrolat**
–	Antennomeres beyond second more or less flattened, antennomeres 4 and 5 wider than apical antennomeres	***Pseudodisonycha* Blake**
4	Head with mid-cranial suture present in lower part of vertex, represented by a short, relatively wide, deep depression. Hind tibia dorsoventrally flattened with grove along its length	***Blakealtica* Viswajyothi & Konstantinov**
–	Head without mid-cranial suture. Hind tibia more or less round in cross section, without grove along its length	**5**
5	Orbit extremely narrow. Frontal ridge long, extending lower than lower side and antennal sockets	***Rosalactica* Bechyne & Bechyne**
–	Orbit generally wide. Frontal ridge does not extend much lower than lower side of antennal sockets	**6**
6	Vertex covered with large closely placed punctations. Elytra often with markings and longitudinal ridges	***Hemilactica* Blake**
–	Vertex covered with small distantly placed punctations. Elytra often without markings, always without longitudinal ridges	**7**
7	Head with transfrontal sulcus absent or poorly impressed. Pronotum mostly with transverse impression. Elytra often with basal callus	***Monomacra* Chevrolat**
–	Head with transfrontal sulcus well impressed. Pronotum mostly without transverse impression. Elytra often without basal callus	***Parchicola* Bechyne & Bechyne**

**Figures 29, 30.**
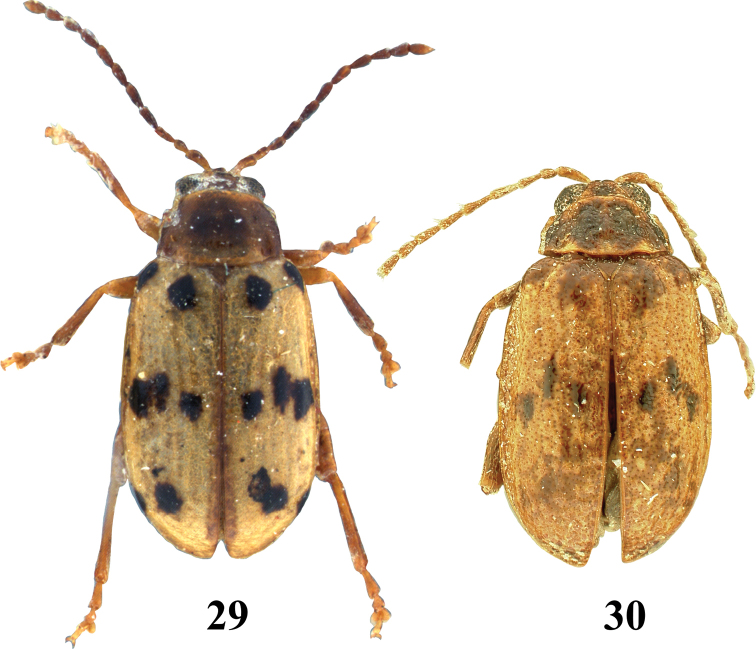
Adult *Hemilactica* species dorsal habitus **29***H.quatuordecimpunctata*, lectotype **30***H.rugosa*, holotype.

## Supplementary Material

XML Treatment for Hemilactica

XML Treatment for Hemilactica erwini

XML Treatment for Hemilactica sierramartingarcia
